# The Role of Endogenous Beta-Endorphin and Enkephalins in the Crosstalk Between Ethanol and Morphine

**DOI:** 10.3390/ph18010107

**Published:** 2025-01-16

**Authors:** Andy Tseng, Syed Muzzammil Ahmad, Abdul Hamid, Kabirullah Lutfy

**Affiliations:** Department of Biotechnology and Pharmaceutical Sciences, College of Pharmacy, Western University of Health Sciences, Pomona, CA 91766, USA; tsenga@westernu.edu (A.T.); smahmad@westernu.edu (S.M.A.); ahamid@westernu.edu (A.H.)

**Keywords:** ethanol, morphine, conditioned place preference, β-endorphin, enkephalins, endogenous opioids, knockout mice, reward, crosstalk

## Abstract

**Background:** There is clinical concern about the combined use of alcohol and opiates. Several lines of evidence support an interaction between alcohol and the endogenous opioid system. Thus, we hypothesized that ethanol, by causing the release of opioid peptides, may sensitize the system to the action of exogenous opioids such as morphine. **Objectives:** In this study, using the place conditioning paradigm, a model of reward, we determined whether a morphine challenge would alter the pre-established preference induced by ethanol conditioning in mice, and whether this response was mediated by the mu opioid receptor (MOP). Given that ethanol exposure stimulates the release of opioid peptides, we also assessed the role of beta-endorphin (β-END) and enkephalins (ENKs) in this response. **Methods:** Mice lacking MOPs, β-END, and/or ENKs, and their respective wild-type controls were tested for preconditioning place preference on day 1. Mice were then conditioned with ethanol (2 g/kg) versus saline on days 2 to 4 and then tested under a drug-free state for postconditioning place preference on day 5. On day 8, mice received a single injection of morphine (5 mg/kg) and were tested for place preference. On the test days, mice were placed in the central chamber and allowed to explore the chambers. The amount of time that mice spent in the drug-paired chamber was recorded. **Results:** We found that a challenge dose of morphine given on day 8 enhanced the conditioned place preference (CPP) response in mice previously conditioned with ethanol. This response was abolished in MOP-null mice, confirming the role of MOPs in this response. Although this enhanced response was not altered in mice lacking either β-END or ENKs compared to their wild-type littermates/controls, it was completely blunted in mice lacking both β-END and enkephalins. **Conclusions:** Together, these results suggest that these opioid peptides jointly mediate the crosstalk between the rewarding actions of morphine and ethanol.

## 1. Introduction

Alcohol is one of the most widely used licit drugs. Notably, it is a powerful reinforcing agent, and its chronic use can lead to addictive behaviors, characterized by the loss of control over intake and, importantly, compulsive alcohol-seeking and alcohol-taking behaviors despite negative health and socioeconomic consequences. However, a limited number of pharmacotherapeutic agents are available to treat this chronic and relapsing brain disorder.

The endogenous opioid system has been implicated in the rewarding and reinforcing actions of alcohol. For example, several studies have shown that nonselective opioid antagonists, such as naloxone and naltrexone, reduce alcohol consumption in both humans and animals [1,2,3,4,5]. There is also evidence that opioid peptides are released following ethanol exposure [6,7,8,9,10]. Furthermore, increased opioid system activity following ethanol exposure in rodent lines selectively bred for alcohol preference has been associated with their propensity to drink relative to non-preferring strains [7,11].

The combined use of alcohol and opiates is also a major medical concern. Cases involving its concomitant use or where it is used as a substitution (e.g., opiate use during periods of abstinence from alcohol, the use of alcohol to cope with pain, or the development of opiate addiction following prior problems with alcohol) have been reported [12]. Numerous studies have shown that a history of alcohol use is a risk factor for increased opioid use. Compared to non-drinkers, binge drinkers were twice as likely to misuse prescription opioids, with the frequency of binge drinking being a strong predictor of misuse [13]. Among those with excessive alcohol use, the proportion expressing chronic pain was highly elevated (>50%), and this was correlated with higher non-prescription opioid use [14,15]. This is a serious issue for people who may consume alcohol while taking pain medications, as the use of these substances together may lead to adverse health consequences such as the enhancement of shared CNS effects (e.g., sedation) as well as a risk of respiratory depression [16]. A toxicologic analysis of victims of heroin overdose found an inverse relationship between blood ethanol and morphine concentrations in the blood, suggesting that the presence of alcohol could alter the acute effects of heroin [17]. Recent reports have estimated that approximately 19% of opioid-related emergency department visits involved alcohol, and 22% of drug-related deaths involved both opioids and alcohol [18]. Given its wide availability, alcohol has often been termed a gateway drug from which users turn to other illicit drugs.

Both alcohol and morphine have been shown to activate a common reward pathway, and each induces euphoria and has a high potential for abuse. Morphine induces its rewarding effect by binding to MOPs, stimulating dopamine release within the mesolimbic reward system. Ethanol targets numerous neurotransmitter systems to exert its rewarding action. Among these, it can facilitate dopaminergic activity by causing the release of opioid peptides, which then act on opioid receptors, leading to its rewarding effects (for review, see [19]). Several studies have also reported an interaction between alcohol and exogenous opioids. For example, there is evidence showing the development of cross-tolerance to the analgesic effect of ethanol in morphine-treated mice [20]. Furthermore, the prior injection of morphine increased the intake of an ethanol solution in rats [21]. In human studies, higher alcohol consumption was associated with altered response to an oral dose of oxycodone indicated by attenuated miosis, poorer performance on cognitive tests, and a higher subjective rating of the positive effects of oxycodone [22]. Together, these studies suggest that changes in drug sensitivity due to the co-use of these substances could lead to altered intake and increased risk for abuse.

We previously found that the rewarding action of ethanol was reduced in mice lacking both beta-endorphin and enkephalins, suggesting that these opioid peptides mediate, at least in part, the rewarding action of alcohol [23]. Here, we propose that if alcohol alters the level of endogenous opioids, exogenously administered opioids should also crosstalk with alcohol. As stated above, previous studies have shown that small doses of morphine increase animals’ intake of alcohol [24]. Given that opioids can also influence consummatory behaviors [25], however, it is difficult to distinguish whether this effect was due to morphine modifying the motivational processes underlying alcohol reward/reinforcement or due to a more nonspecific effect on feeding. Therefore, we used the place conditioning paradigm as a measure of reward [26], and assessed if ethanol-induced conditioned place preference (CPP) would display crosstalk with morphine. We then determined the role of MOPs in this response, in which we compared mu opioid receptor (MOP) knockout mice to their wild-type littermates/controls. This was to confirm that the MOP mediates morphine’s actions. We then evaluated which endogenous opioid peptide(s) would be involved in the crosstalk between alcohol and morphine, in which we utilized mice lacking β-END and/or enkephalins and their wild-type controls.

## 2. Results

### 2.1. Ethanol-Induced CPP in C57BL/6J Mice, and This Response Was Potentiated by a Morphine Challenge

**Figure 1:**
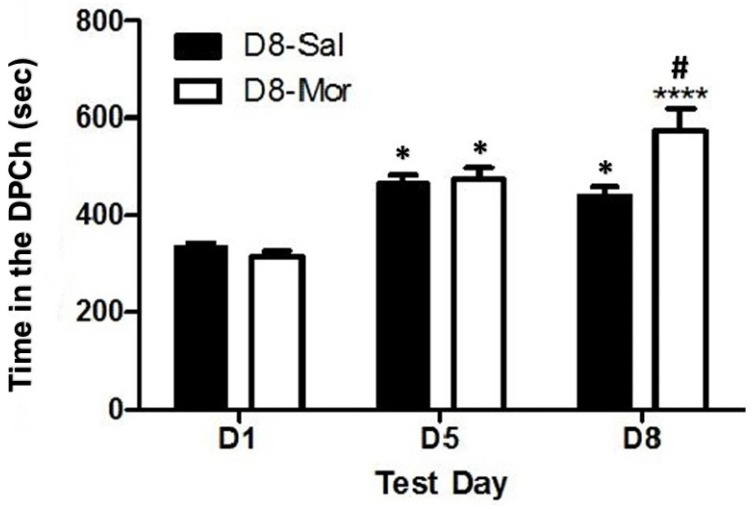
Ethanol-induced CPP in mice, and this response was enhanced by morphine. Female C57BL/6J mice were tested for baseline on day 1 (D1), conditioned with ethanol (2 g/kg)/saline or saline/ethanol twice daily on days 2–4, tested for CPP on day 5 (D5) and then on day 8 (D8) following a saline (D8-Sal) or morphine (5 mg/kg; D8-Mor) challenge. Data are mean (±SEM) of the amount of time that mice (23–30 mice per group) spent in the drug-paired chamber (DPCh) on the preconditioning day (D1), postconditioning day (D5) and challenge day (D8). * *p* < 0.05 versus D1 for each group; **** *p* < 0.0001 vs. D1; # *p* < 0.05 vs. D8-Sal.

Figure 1 depicts the amount of time that C57BL/6J mice spent in the ethanol-paired chambers before (D1) and after (D5) conditioning with ethanol (2 g/kg) and following the saline (D8-Sal) or morphine (D8-Mor) challenge given on day 8 (D8). Two-way repeated measures ANOVA revealed no significant effect of treatment (F(1,51) = 3.59, *p* > 0.05), but there was a significant effect of test day (F(2,102) = 51.32, *p* < 0.0001) and a significant interaction between the two factors (F(2,102) = 9.26, *p* < 0.0005). Post hoc analysis showed that mice spent significantly more time in the ethanol-paired chamber on the postconditioning test day (day 5, D5) than on the preconditioning test day (day 1, D1), showing that both groups exhibited CPP. Animals challenged with saline on day 8 retained the CPP response. However, mice that were treated with morphine on day 8 spent a significantly greater amount of time in the ethanol-paired chamber than their saline-treated controls (*p* < 0.0001). These results suggest that morphine potentiated the rewarding action of ethanol in wild-type mice (Figure 1).

### 2.2. Morphine Potentiated Ethanol-Induced CPP in Wild-Type but Not MOP-Null Mice

**Figure 2:**
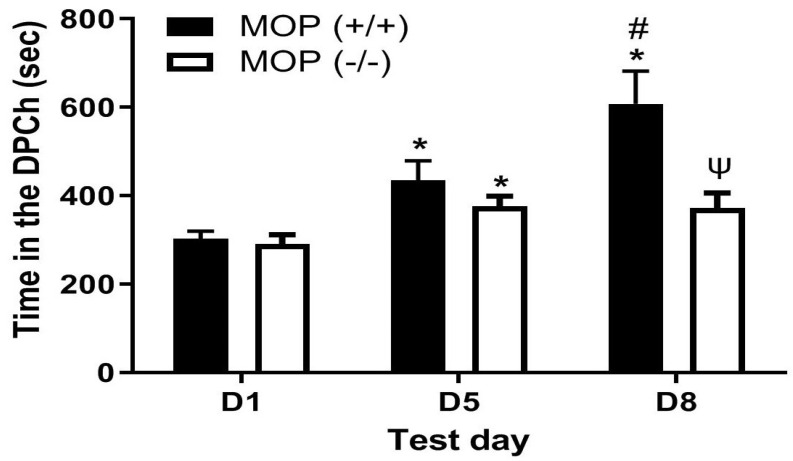
Mice lacking MOPs (MOP−/−) showed a comparable CPP to their wild-type control (MOP+/+) following ethanol conditioning but failed to show the enhanced response following the morphine challenge. Mice were tested for baseline place preference on day 1 (D1), conditioned with ethanol (2 g/kg)/saline or saline/ethanol twice daily on days 2–4, tested for CPP on day 5 (D5) and then on day 8 (D8) following a morphine (5 mg/kg) challenge. Data are mean (±SEM) the amount of time that mice (6–9 mice per genotype) spent in the drug-paired chambers (DPCh) on preconditioning (D1) and postconditioning (D5) test days and following a morphine challenge (D8). * *p* < 0.05 vs. D1 for each genotype, # *p* < 0.05, D8 vs. D5 in MOP+/+ mice; ψ *p* < 0.05, MOP−/− vs. MOP+/+ on D8.

Figure 2 depicts the amount of time that mice lacking MOPs and their wild-type littermates/controls spent in the ethanol-paired chambers before (D1) and after (D5) conditioning with ethanol (2 g/kg) and following a challenge dose of morphine (5 mg/kg) given on day 8 (D8). Individual data points on the bar chart are provided in the Supplemental Materials (Figure S1). Comparing the amount of time that the mice of both genotypes spent in the ethanol-paired chamber on pre- and postconditioning test days revealed a significant effect of test day (F(2,26) = 15.17, *p* < 0.001) and a significant effect of genotype (F(1,13) = 9.49 (*p* < 0.01) and a significant interaction between the two factors (F(2,26) = 5.64, *p* < 0.01). Post hoc analysis showed that wild-type mice spent a significantly greater amount of time in the ethanol-paired chamber on day 8 compared to MOP-null mice (*p* < 0.05). These results suggest that, although ethanol induced CPP in both mice lacking MOPs and their wild-type littermates/controls (Figure 2, compare D1 vs. D5), the CPP response following a challenge dose of morphine was attenuated in MOP-null mice (Figure 2, compare the CPP response in mice of the two genotypes on D8). These findings suggest that the ability of morphine to enhance the magnitude of ethanol CPP was mediated via MOPs.

### 2.3. Morphine-Induced Potentiation of Ethanol CPP Was Not Altered in Mice Lacking β-END Compared to Wild-Type Controls

**Figure 3:**
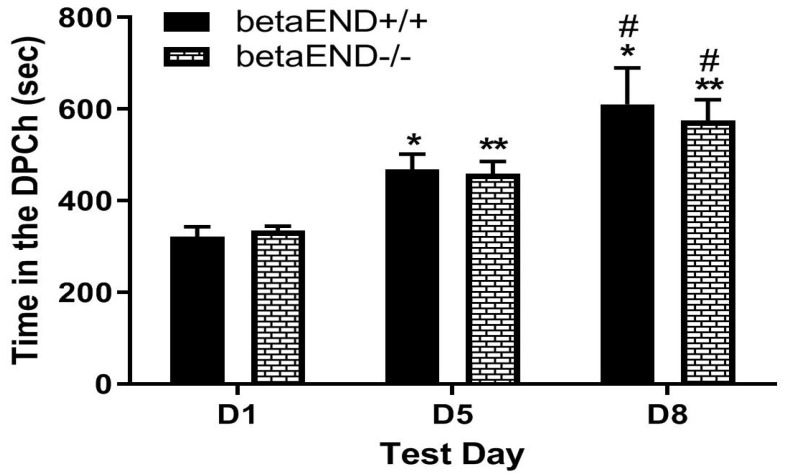
Ethanol induced a comparable CPP in mice lacking β-END (betaEND−/−) and their wild-type controls (betaEND+/+) under a drug-free state on day 5 and following the morphine challenge on day 8. Mice lacking beta-END and their wild-type littermates/age-matched controls were tested for preconditioning place preference on day 1 (D1), conditioned with ethanol (2 g/kg)/saline or saline/ethanol twice daily on days 2–4, tested for CPP on day 5 (D5) and then on day 8 (D8) following a morphine (5 mg/kg) challenge. Data are mean (±SEM) of the amount of time that mice (6–7 mice per genotype) spent in drug-paired chambers (DPCh) on the preconditioning (D1) and postconditioning (D5) test days and following a morphine challenge (D8) in null mice. * *p* < 0.05 ** *p* < 0.01 vs. D1 for each genotype, # *p* < 0.05 versus D5 for each genotype.

Figure 3 depicts the amount of time that mice lacking β-END spent in the ethanol-paired chambers before (D1) and after (D5) conditioning with ethanol (2 g/kg) and following a challenge dose of morphine (5 mg/kg) given on day 8 (D8). Individual data points on the bar chart are provided in the Supplemental Materials (Figure S2). Two-way repeated measures ANOVA revealed a significant effect of test day (F(2,22) = 31.73, *p* < 0.0001), but no significant effect of genotype (F(1,11) = 0.06, *p* > 0.05) and no significant interaction between the two factors (F(2,22) = 0.27, *p* > 0.05). However, there was no significant difference between the mice of the two genotypes in the amount of time that they spent in the ethanol-paired chamber (DPCh) on the preconditioning (D1) and postconditioning (D5) test days (*p* > 0.05). Likewise, the mice of both genotypes showed a comparable CPP response following the challenge dose of morphine on day 8 (Figure 3, D1 vs. D8 for the mice of each genotype). These results suggest that the ability of morphine to potentiate ethanol CPP was not altered in the absence of β-END.

### 2.4. Morphine-Induced Potentiation of Ethanol CPP Was Not Altered in Mice Lacking Enkephalins Compared to Wild-Type Controls

**Figure 4:**
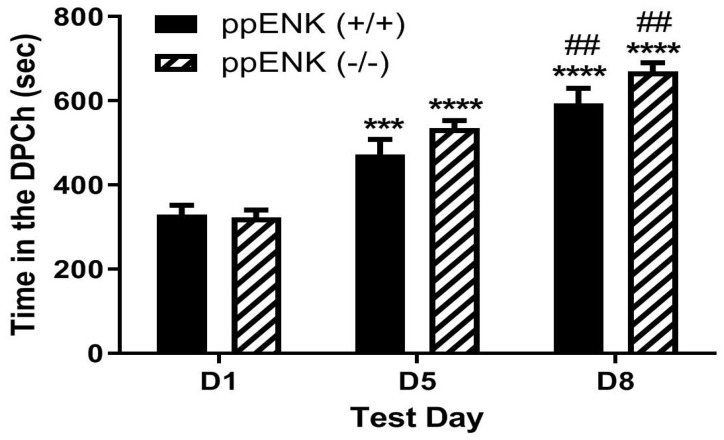
The CPP response was comparable in mice lacking the *preproENK* gene (ppENK−/−) and their wild-type controls on day 5 and after the morphine challenge on day 8. Mice lacking the ppENK gene and their wild-type littermates/age-matched controls were tested for baseline place preference on day 1 (D1), conditioned with ethanol (2 g/kg)/saline or saline/ethanol twice daily on days 2–4, tested for CPP on day 5 (D5) and then on day 8 (D8) following a morphine (5 mg/kg) challenge. Data represent the amount of time that mice spent in ethanol-paired chambers on the preconditioning (D1) and postconditioning (D5) test days and following a morphine challenge (D8) in mice of the two genotypes. *** *p* < 0.001, **** *p* < 0.0001 versus D1 for each genotype; ## *p* < 0.01 vs. D5 for each genotype.

Figure 4 depicts the amount of time that mice lacking enkephalins spent in the ethanol-paired chambers before (D1) and after (D5) conditioning with ethanol (2 g/kg) and following a challenge dose of morphine (5 mg/kg) given on day 8 (D8). Individual data points on the bar chart are provided in the Supplemental Materials (Figure S3). Two-way repeated measures ANOVA revealed a significant effect of test day (F(2,24) = 65.74, *p* < 0.0001), but there was no significant effect of genotype (F(1,12) = 2.74, *p* > 0.05) and no significant interaction between the two factors (F(2,24) = 1.39, *p* > 0.05). However, there was no difference in the amount of time that mice lacking enkephalins spent in the ethanol-paired chamber (DPCh) compared to wild-type controls on the preconditioning and postconditioning test days (*p* > 0.05). In addition, there was no difference between genotypes in the amount of time spent in the ethanol-paired chamber on day 8 (Figure 4, D1 vs. D8). These results suggest that the ability of morphine to potentiate ethanol CPP was not altered in the absence of enkephalins.

### 2.5. Morphine-Induced Potentiation of Ethanol CPP Was Abolished in Mice Lacking Both β-END and Enkephalins

**Figure 5:**
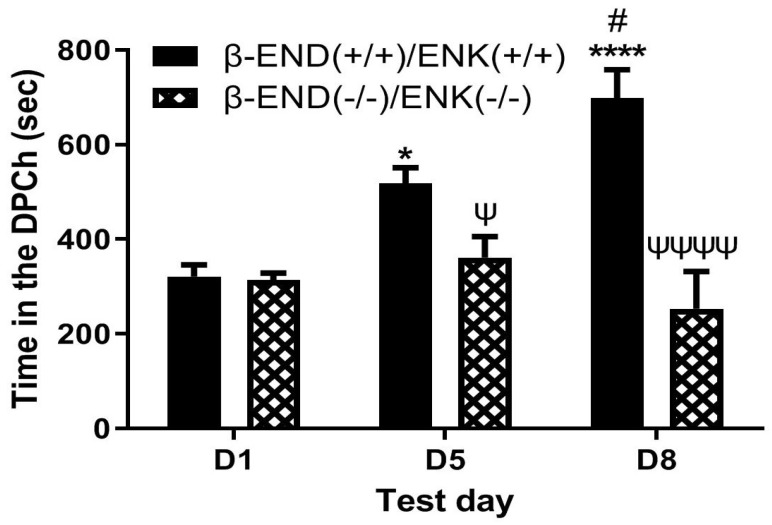
Mice lacking β-END and ENK failed to exhibit CPP following ethanol conditioning and after the morphine challenge compared to their wild-type controls [β-END(+/+)/ENK(+/+)]. Mice lacking both β-END and ENKS and their wild-type littermates/age-matched controls were tested for baseline place preference on day 1 (D1), conditioned with ethanol (2 g/kg)/saline or saline/ethanol twice daily on days 2–4, tested for CPP on day 5 (D5) and then on day 8 (D8) following a morphine (5 mg/kg) challenge. Data represent mean (±SEM) of the amount of time that mice (*n* = 6 mice per genotype) spent in the drug-paired chamber (DPCh) on preconditioning (D1) and postconditioning (D5) test days and following a morphine challenge (D8) in null mice. * *p* < 0.05, **** *p* < 0.0001 versus D1 for double wild-type mice. # *p* < 0.05, vs. D5 in wild-type mice; ^ψ^ *p* < 0.05, ^ψψψψ^ *p* < 0.0001; a significant decrease in double knockout mice than double wild-type mice on D5 and D8, respectively.

Figure 5 depicts the amount of time that mice lacking β-END and enkephalins spent in the ethanol-paired chambers before (D1) and after (D5) conditioning with ethanol (2 g/kg) and following a challenge dose of morphine (5 mg/kg) given on day 8 (D8). Individual data points on the bar chart are provided in the Supplemental Materials (Figure S4). Two-way repeated measures ANOVA revealed a significant effect of test day (F(2,20) = 5.56, *p* < 0.01) and a significant effect of genotype (F(1,10) = 31.67, *p* < 0.001), and a significant interaction between the two factors (F(2,36) = 9.99, *p* < 0.001). Post hoc analysis showed that there was no difference in the time that mice lacking β-END and enkephalins spent in the ethanol-paired chamber on the preconditioning test day (*p* > 0.05) compared to wild-type controls. However, wild-type mice spent a significantly greater amount of time in the ethanol-paired chamber on the postconditioning test day compared to the double knockout mice (*p* < 0.05) (Figure 5, D1 vs. D5). These results confirm those previously shown, that the rewarding action of ethanol was reduced in mice lacking β-END and enkephalins compared to wild-type mice. Post hoc analysis also showed that wild-type controls spent a significantly greater amount of time in the ethanol-paired chamber on day 8 (*p* > 0.05) than the double knockout mice (Figure 5, compare mice of the two genotypes on D8). These results suggest that the ability of morphine to potentiate the CPP response was abolished in the absence of both β-END and enkephalins.

## 3. Discussion

The main finding of the present study is that there was a crosstalk between the rewarding actions of morphine and ethanol, as a challenge dose of morphine enhanced the CPP response in mice conditioned with ethanol. This was blunted in mice lacking the MOP, confirming that the response is mediated via the MOP. While the enhancement was not altered in mice lacking β-END or enkephalins alone, it was completely abolished in mice lacking both β-END and enkephalins. Together, these results suggest that the joint action of these opioid peptides on ethanol reward plays a critical role in the crosstalk between morphine and ethanol.

The prevalence of multiple substance abuse, in particular involving alcohol and opiates, gives reason to investigate the mechanisms underlying their combined use. Alcohol and morphine produce many similar effects, such as euphoria, and have overlapping mechanisms of action. Morphine mimics the action of endogenous opioids by binding to the MOP. Alcohol, on the other hand, stimulates the release of endogenous opioid peptides, such as β-END and enkephalins, in turn activating opioid receptors [6,7,8,9,27]. Morphine has been shown to suppress ethanol withdrawal-induced convulsions in mice [28], while sensitization to morphine developed in mice that were habituated to alcohol [29]. These studies suggest that there is a crosstalk between morphine’s and alcohol’s effects. A correlation has also been observed between morphine and alcohol consumption, as small doses of morphine have been shown to increase the subsequent intake of ethanol in rats [24]. However, the ability of morphine to alter alcohol reward has not been fully explored. Therefore, we used the place conditioning paradigm to assess whether morphine would alter ethanol-induced CPP.

Animals were conditioned with ethanol (2 g/kg), tested for postconditioning CPP on day 5 and then received a challenge dose of morphine (5 mg/kg) on day 8. The choice of the dose of ethanol and the selection of female mice were based on our previous studies, as this dose of ethanol induced a robust CPP response in female mice [23,30,31]. We found that ethanol conditioning induced a robust CPP in wild-type mice of all genotypes, and a subsequent morphine challenge potentiated the CPP response in these mice. These results are in agreement with earlier studies showing that pretreatment with morphine following ethanol-induced CPP leads to the persistent expression of the CPP response [32].

Previous studies have assessed the interaction between ethanol and opioids. For example, morphine-induced CPP was potentiated in mice given a liquid diet containing ethanol, supporting an interaction between alcohol and opioid effects [33]. Furthermore, binge-like exposure to ethanol upregulated MOP expression in the striatum and nucleus accumbens, leading to an increase in the antinociceptive response to morphine [34]. Given that the action of morphine is mediated via MOPs, we also examined the role of MOPs in the crosstalk between morphine and ethanol reward. We observed that the MOP wild-type mice conditioned with ethanol and challenged with morphine exhibited a greater CPP response, and this response was absent in mice lacking MOPs, suggesting that the crosstalk between morphine and alcohol reward is dependent on a common mechanism in the CNS, which most likely involves the activation of MOPs. Ethanol exposure not only alters the release of endogenous opioid peptides but also opioid receptor expression along mesolimbic dopaminergic neurons [35,36]. Thus, the upregulation of MOPs following alcohol exposure may lead to a sensitized endogenous opioid system, permitting enhanced opioid receptor binding. This, in turn, would facilitate the effect of a subsequent morphine challenge, thereby leading to a greater CPP response. Alternatively, given that both alcohol and morphine alter dopamine release, the enhancement of reward may be due to a sensitized dopamine system. For example, voluntary ethanol consumption increased the number of spontaneously firing dopamine neurons in the VTA, which would lead to an excitatory effect on dopamine release [37]. While additional research is required to identify the neuroanatomical site of this interaction, we predict that the VTA could be at least one of the sites of this interaction, as opioids are thought to cause the disinhibition of mesolimbic dopaminergic neurons [38] and the VTA is the site for the development of sensitization [39]. Furthermore, dose–response and time-course studies are needed to define the range of doses that elicit this crosstalk and how long-lasting this crosstalk would be. Additionally, it would be interesting to assess whether this crosstalk could occur using other opioids as well as other classes of addictive drugs.

Given that β-END and enkephalins bind to the MOP and that opioid peptides are released following ethanol exposure [9,10], we hypothesized that the ability of morphine to enhance the rewarding action of ethanol might be due to the release of endogenous opioid peptides during ethanol conditioning. Therefore, this response would be altered in the absence of β-END or enkephalins. However, we discovered that mice lacking either β-END or enkephalins exhibited a comparable CPP response compared to wild-type controls following the ethanol conditioning and in response to the morphine challenge. Because we had previously observed reduced ethanol CPP in mice lacking both peptides [23], suggesting that one peptide might compensate for the other, we then examined whether the morphine-induced potentiation of ethanol CPP would be altered in mice lacking both β-END and enkephalins. Our results showed that morphine enhanced the rewarding action of ethanol in wild-type mice. However, the crosstalk was absent in mice lacking both β-END and enkephalins, suggesting that both β-END and enkephalins are needed for the development of crosstalk between ethanol and morphine. We propose that the release of endogenous opioid peptides by ethanol [6,7,8,27,40] may induce a sensitized response, thereby leading to enhanced CPP following the morphine challenge in mice. An alternative explanation is that because double knockout mice did not exhibit CPP on day 5, they did not express CPP on day 8 following the morphine challenge. However, we found that mice showing no CPP following conditioning with a low dose of morphine (5 mg/kg, s.c.) exhibited a robust state-dependent CPP following the same challenge dose of morphine [41]. Nevertheless, further studies are needed to differentiate between these two possibilities and to delineate the underlying mechanisms of the crosstalk. Based on the current data, we propose that ethanol causes the release of opioid peptides during conditioning, leading to CPP and cross-sensitization to opioids, such as morphine. A direct interaction of morphine with mu opioid receptors is necessary for the crosstalk, as we did not observe this phenomenon in mice lacking MOPs. However, future studies are needed to define whether the changes are due to a direct action of morphine on the MOP or due to downstream changes occurring following the stimulation of the MOP by morphine.

The role of the delta opioid receptor (DOP) in alcohol reward has been explored using antagonists with increased selectivity for this receptor, which have yielded mixed results. For example, the DOP1-selective agonist TAN-67 enhanced ethanol-induced CPP while the DOP2-selective agonist SNC-80 completely abolished the expression of place preference induced by ethanol [42]. Likewise, while alcohol intake was enhanced in mice lacking the delta opioid receptor [43], mice lacking enkephalins showed no change in alcohol consumption [44]. Furthermore, mice lacking enkephalin and their wild-type controls showed a comparable CPP [45] following conditioning with ethanol (2 g/kg), a dose that was used in the present study. Therefore, we believe that the delta receptors may not play a significant role in the crosstalk observed between alcohol and morphine. However, it may be that the crosstalk is mediated via the DOP-MOP dimers [46]. Future studies should be specifically tailored to investigate the latter possibility.

## 4. Materials and Methods

### 4.1. Subjects

Female mice between the ages of 2 and 4 months at the time of the experiments were used throughout. We used female mice because they exhibit a more robust CPP response than male mice following an ethanol conditioning protocol similar to that used in the current study [30]. Mice lacking MOPs [47], β-endorphin [48], and enkephalin [49], and their respective wild-type controls, fully backcrossed on a C57BL/6J mouse background, originally obtained from the Jackson Laboratory (Bar Harbor, ME, USA) were bred in house. Male and female heterozygous mice of each genotype or both lines were generated and then mated to obtain mice lacking β-endorphin, enkephalins or β-endorphin and enkephalins, and their respective wild-type littermates/controls. Mice were housed up to 4 per cage, with food and water available *ad libitum* throughout the study. They were kept in a temperature- and humidity-controlled room with a standard 12 h light/dark cycle. All experiments were conducted during the light phase between 9:00 AM to 4:00 PM. Mice were habituated to the testing room at least 1 day prior to the start of the experiment and remained there until the end of the experiment. All procedures were in accordance with the National Institute of Health (NIH) Guidelines for the Care and Use of Laboratory Animals (IACUC) and approved by the Institutional Animal Care and Use Committee (R13/IACUC/022) at Western University of Health Sciences (Pomona, CA, USA).

### 4.2. Drugs

Alcohol solutions were prepared from ethyl alcohol (200 Proof, OmniPur, EM Science; Gibbstown, NJ, USA) and saline to the appropriate concentrations, and injected intraperitoneally (i.p.) in a volume of 0.1 mL/10 g body weight. Morphine, generously supplied by the drug supply program of the National Institute on Drug Abuse (NIDA), was dissolved in saline and injected subcutaneously (s.c.) in a volume of 0.1 mL/10 g body weight.

### 4.3. Place Conditioning Paradigm

#### 4.3.1. Apparatus

A 3-chambered place conditioning apparatus (ENV-3013, Med Associates Inc., St. Albans, VT, USA) was used. The apparatus was divided into two large equal-sized chambers (16.8 × 12.7 × 12.7 cm), connected by a small central chamber (7.2 × 12.7 × 12.7 cm), and separated by manual guillotine doors. The two side chambers contained distinct visual and tactile cues. One chamber had a white wall with horizontal or vertical black stripes and steel mesh floors, while the opposite chamber had a black wall with horizontal or vertical white stripes and solid steel rod floors. The central neutral chamber had solid gray walls and a solid gray floor and served as the entry point to the apparatus. The apparatus was equipped with infrared beams connected to a computer system with software (MED-PC IV, ENV-3013, Med Associates Inc. (St. Albans, VT, USA) to monitor and record the amount of time that mice spent in each chamber.

#### 4.3.2. Experimental Procedure

**Figure 6:**
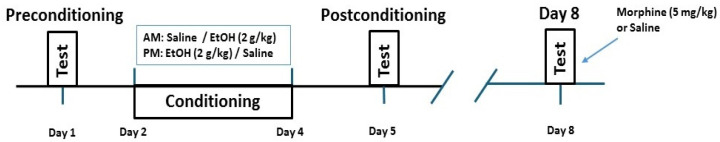
Schematic diagram of the experimental procedure for the place conditioning paradigm.

The place conditioning procedure consisted of three distinct phases conducted over 5 days: preconditioning (day 1), conditioning with saline or ethanol (days 2–4), and postconditioning (day 5). On the preconditioning test day, mice were tested for baseline place preference, in which they were placed in the central neutral gray chamber and allowed to explore the entire apparatus for 15 min under a drug-free state. The amount of time that mice spent in each conditioning chamber was recorded. Conditioning was conducted using a biased design, in which mice received ethanol (2 g/kg) in the initially non-preferred side and saline in the initially preferred side in a counterbalanced manner. Every attempt was made to include knockout mice with their respective wild-type controls in each set of animals undergoing testing and conditioning. Additionally, each experiment was conducted using at least three cohorts of 3–5 mice per genotype. During the conditioning days (days 2–4), animals received an injection of ethanol (2 g/kg, i.p.) or saline in the morning and were immediately confined to the corresponding ethanol- or saline-paired chamber for 15 min. In the afternoon, they received the alternate treatment and were confined to the opposite chamber for 15 min. The morning and afternoon conditioning sessions were separated by 4 h. On the postconditioning test day, mice were again placed in the central chamber and allowed to explore the entire apparatus for 15 min. The time that mice spent in each chamber was recorded in the same manner as for the preconditioning test. Mice were left undisturbed in their home cages over the next 2 days. The selection of ethanol dose (2 g/kg, i.p.) was based on our earlier studies [30,31]. On day 8, mice were challenged with morphine (5 mg/kg, s.c.) and tested for CPP again. The schematic diagram of the experimental procedure is shown below (Figure 6).

**Experiment 1. To determine if mice conditioned with ethanol exhibit altered reward in response to a morphine challenge.** We determined if mice conditioned with ethanol would display greater CPP when challenged with morphine. Mice were conditioned as described above and on day 8 tested for CPP following saline or a challenge dose of morphine (5 mg/kg, s.c.). Mice were then immediately placed in the place conditioning apparatus. The amount of time that mice spent in each chamber was measured for 15 min. The choice of morphine dose (5 mg/kg, s.c.) was based on our published [41] and pilot studies (Hamid and Lutfy, unpublished data).

**Experiment 2. To assess the role of the mu opioid receptor (MOP) in the crosstalk between ethanol and morphine.** We then evaluated the role of the MOP in this response, in which we compared MOP knockout mice to their wild-type littermates/controls. Mice were conditioned as described above and, on day 8, tested for CPP following a challenge dose of morphine (5 mg/kg, s.c.). The amount of time that mice spent in each chamber was measured for 15 min.

**Experiment 3. To investigate the role of beta-endorphin and/or enkephalins in the crosstalk between ethanol and morphine.** We then evaluated which endogenous opioid peptides would be involved in the crosstalk between alcohol and morphine. Mice lacking β-END and/or enkephalins and their wild-type controls were conditioned with ethanol, as described above. On day 8, they were challenged with morphine (5 mg/kg, s.c.) and immediately after were placed in the place conditioning apparatus. The time that mice spent in each chamber was recorded for 15 min.

### 4.4. Data Analysis

Data are expressed as mean (±SEM) of the amount of time that mice of each genotype spent in the ethanol-paired chambers on preconditioning (D1) test day, following conditioning with ethanol (2 g/kg) (D5) and following a challenge dose of morphine (5 mg/kg) (D8). Data were analyzed using repeated measures analysis of variance (ANOVA). Fisher’s LSD post hoc test was used to reveal significant differences between groups and between test days. A *p* < 0.05 was considered significant. GraphPad Prism Software, Version 9 (San Diego, CA, USA) was used to analyze the data and create the figures.

## 5. Conclusions

In the present study, we observed that morphine potentiated the rewarding properties of ethanol, and this effect was blocked in MOP-null mice as well as in mice lacking both β-end and enkephalins. Therefore, it seems that the interaction between the rewarding effects of morphine and alcohol results from a convergence in their mechanisms, potentially through the ethanol-induced release of opioid peptides activating MOPs, which could sensitize the system to an exogenous opioid, such as morphine. Additional research is required to explore this hypothesis further. Intriguingly, chronic ethanol drinking has been shown to produce tolerance to morphine’s analgesic effects by preventing MOP endocytosis [50]. Consequently, these findings may have important implications, especially in alcoholics needing morphine for pain relief. Since cross-tolerance to the analgesic effects and cross-sensitization to the rewarding action of ethanol and morphine occur, consideration needs to be given when pain therapy is necessary in these patients.

## Data Availability

Data will be made available upon reasonable request.

## Supplementary Materials

The following supporting information can be downloaded at: https://www.mdpi.com/article/10.3390/ph18010107/s1, Figure S1: Ethanol CPP (D5) and crosstalk (D8) between morphine and ethanol in mice lacking MOP and their wild-type controls; Figure S2: Ethanol CPP (D5) and crosstalk (D8) between morphine and ethanol in mice lacking beta-endorphin and their wildtype controls; Figure S3: Ethanol CPP (D5) and crosstalk (D8) between morphine and ethanol in mice lacking the *ppENK* gene and their wildtype controls; Figure S4: Ethanol CPP (D5) and crosstalk (D8) between morphine and ethanol in mice lacking both beta-endorphin and enkephalins and their wildtype controls.
