# Supplementary material for: The Role of Endogenous Beta-Endorphin and Enkephalins in the Crosstalk Between Ethanol and Morphine
Source: Pharmaceuticals (Basel). 2025 Jan 16;18(1):107. doi: 10.3390/ph18010107 (PMC11768102; doi:10.3390/ph18010107)
Supplement: Supplementary file 1 [file pharmaceuticals-18-00107-s001.zip › pharmaceuticals-3368619-supplementary.pdf]

Supplemental Data

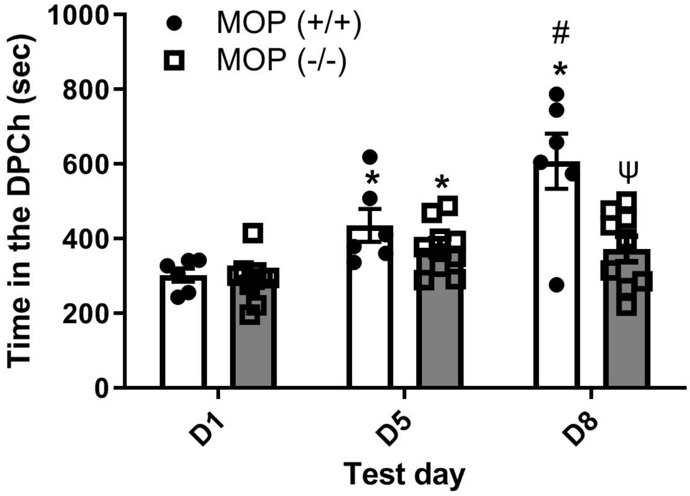

Figure S1: Ethanol CPP (D5) and crosstalk (D8) between morphine and ethanol in mice lacking MOP and their wild-type controls

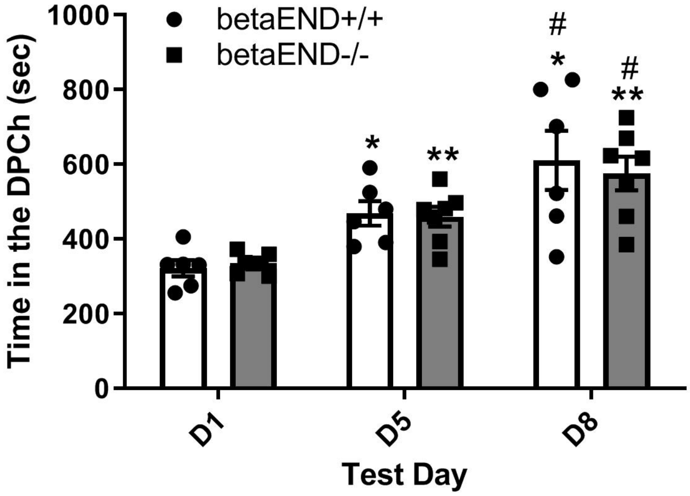

Figure S2: Ethanol CPP (D5) and crosstalk (D8) between morphine and ethanol in mice lacking beta-endorphin and their wildtype controls

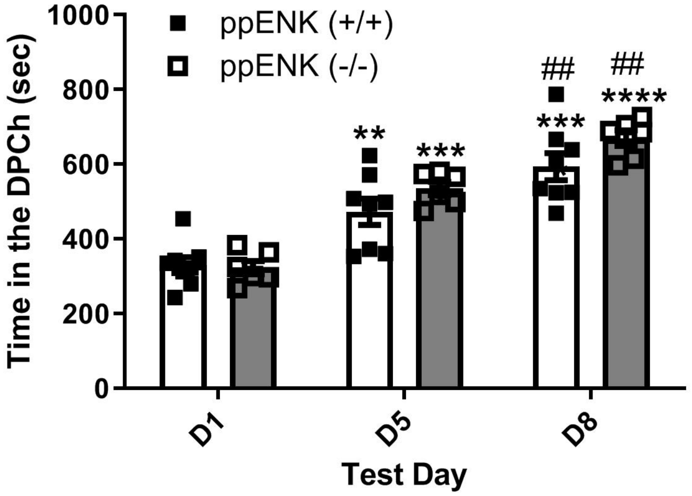

Figure S3: Ethanol CPP (D5) and crosstalk (D8) between morphine and ethanol in mice lacking the *ppENK* gene and their wildtype controls

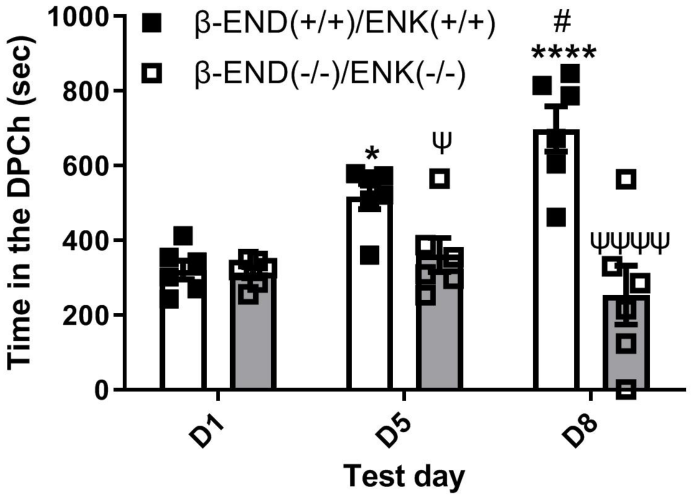

Figure S4: Ethanol CPP (D5) and crosstalk (D8) between morphine and ethanol in mice lacking both beta-endorphin and enkephalins and their wildtype controls
